# Melanoma in Women of Reproductive Age: From Awareness and Prevention to Pregnancy-Associated Management

**DOI:** 10.3390/cancers17213528

**Published:** 2025-10-31

**Authors:** Martyna Różańska, Karolina Orda, Agata Góral, Karolina Niewola, Magdalena Łyko, Alina Jankowska-Konsur

**Affiliations:** 1 Student Research Group of Experimental Dermatology, University Centre of General Dermatology and Oncodermatology, Wroclaw Medical University, Borowska 213, 50-556 Wrocław, Poland; 2 University Centre of General Dermatology and Oncodermatology, Wroclaw Medical University, Borowska 213, 50-556 Wrocław, Poland; alina.jankowska-konsur@umw.edu.pl

**Keywords:** pregnancy-associated melanoma, women of reproductive age, photoprotection, melanocytic nevi, immunotherapy

## Abstract

**Simple Summary:**

Melanoma is one of the most common cancers diagnosed in young women, and its occurrence during pregnancy creates unique challenges for both patients and doctors. While many women protect their skin better than men, important gaps remain in awareness and preventive behaviors, especially among women from different ethnic backgrounds. Pregnancy can cause visible changes in moles, which may worry women and complicate diagnosis. At the same time, safe protection from the sun is essential, with special attention to mineral-based sunscreens and simple lifestyle measures such as seeking shade and wearing protective clothing. Most diagnostic and surgical procedures are safe during pregnancy, but treatment of advanced melanoma requires careful balancing of benefits and risks for both mother and baby. This review highlights the importance of education, prevention, and multidisciplinary care to improve outcomes for women of reproductive age who face the risk of skin cancer.

**Abstract:**

Melanoma poses a growing concern for women of reproductive age, especially during pregnancy, when physiological changes can influence disease presentation and management. While women generally engage more in protective behaviors than men, significant disparities in awareness and preventive practices persist, particularly among racial and ethnic minorities. Pregnancy-associated hormonal and mechanical changes may alter the morphology of nevi, necessitating vigilant dermatologic monitoring. Photoprotection strategies should prioritize safety for both mother and fetus, emphasizing mineral-based sunscreens and behavioral interventions. Although standard diagnostic and treatment approaches remain broadly applicable during pregnancy, advanced melanoma presents unique therapeutic challenges. Targeted therapies like BRAF inhibitors may offer maternal benefits in selected cases, although data are limited, whereas immunotherapies require cautious consideration due to potential fetal risks. Ultimately, optimal care of melanoma during pregnancy demands a multidisciplinary approach that integrates dermatologic, obstetric, oncologic, and pediatric expertise to balance maternal health needs with fetal safety. This review addresses awareness and preventive behaviors in women of reproductive age, pregnancy-related changes in melanocytic nevi, and management considerations during pregnancy.

## 1. Introduction

In recent years, the incidence of melanoma has continued to rise, with approximately one in three affected women being of reproductive age [1,2]. Pregnancy-associated melanoma (PAM) is generally defined as a melanoma identified during pregnancy or within the 12 months postpartum. Earlier studies conducted in the pre-immunotherapy era suggested that PAM is linked with less favorable outcomes than melanoma diagnosed outside of pregnancy [3]; however, more recent analyses are mixed and, after accounting for stage and other confounders, some do not demonstrate materially worse survival. Given these conflicting data, it is prudent to state that the prognostic impact of pregnancy on melanoma remains uncertain and likely influenced by multiple biases [4]. As the incidence of cutaneous melanoma rises among fair-skinned populations, more cases are being diagnosed in women who are pregnant or planning to conceive [2]. Improved screening and heightened awareness have also contributed to increased detection rates in this group. Global trends indicate a steady shift toward later maternal age, driven by educational, professional, and lifestyle factors. Some observational studies have reported that delayed childbearing may be associated with a higher risk of cutaneous malignant melanoma (e.g., an approximate 16% increase per 5-year delay in age at first birth [4,5,6]. However, recent evidence is inconsistent after adjusting for ultraviolet exposure and other confounders. These trends underscore the need for prevention, early detection, and management strategies specifically tailored to women of reproductive age.

This review aims to fill the existing knowledge gap and summarize current evidence regarding awareness and preventive behaviors during pregnancy and in women of reproductive age, examine the impact of pregnancy on melanocytic nevi, and outline key considerations in the diagnosis and management of melanoma during pregnancy. By focusing on this specific population, we hope to contribute to a more comprehensive understanding of melanoma risk and care in women during their reproductive years. The literature for this narrative review was identified through searches of the PubMed and Google Scholar databases, limited to English-language publications. Search terms included combinations of the following keywords: skin cancer awareness, melanoma awareness, melanoma, nevi, moles, melanocytic lesions, pregnant, pregnancy, photoprotection, BRAF inhibitors, and immunotherapy.

## 2. Melanoma Awareness in Women of Reproductive Age

Awareness of melanoma among women of reproductive age has become an important public health concern, as the incidence of this malignancy is rising most rapidly in females under 45 years of age [7]. Although women generally report greater attention to skin health and preventive behaviors, significant knowledge gaps and behavioral inconsistencies remain [8,9,10]. Understanding gender-related differences, sociocultural influences, and disparities in awareness is essential to designing effective preventive strategies [7].

### 2.1. General Awareness and Preventive Behaviors

Women of reproductive age tend to demonstrate higher overall awareness of sun safety compared with men. They more frequently report using sunscreen, seeking shade, and avoiding deliberate exposure to ultraviolet (UV) radiation and sunburn [8,9,10]. In an Australian study, women were twice as likely as men to use sunscreen regularly and significantly more likely to apply it daily [11]. Despite this apparent advantage, misconceptions persist, such as the belief that sunscreen alone offers complete protection or that short exposures do not accumulate risk over time.

### 2.2. Gender Differences and Early Detection

Early detection of melanoma substantially improves prognosis, and women often display behaviors that favor earlier recognition. They tend to pay closer attention to the condition of their skin, particularly the face and extremities—common anatomical sites for melanoma presentation [12]. Furthermore, women make more frequent use of healthcare services, which creates more opportunities for preventive counseling and clinical skin examinations [13]. These gender-related patterns may partly explain why melanomas in women are often diagnosed at a thinner stage compared with those in men.

### 2.3. Racial and Ethnic Disparities in Awareness

Significant disparities exist among racial and ethnic groups regarding awareness of melanoma risk. Many Black and Hispanic women perceive their darker skin as protective and do not consider themselves at significant risk of skin cancer [14]. In these populations, sun-protective practices are often motivated by cosmetic rather than health-related reasons—most commonly to prevent unwanted pigmentation or photoaging. This misconception, combined with limited access to dermatologic care, contributes to delayed diagnosis and poorer outcomes. Addressing these inequities requires culturally tailored educational initiatives that explicitly highlight melanoma risk in individuals of all skin types.

### 2.4. Indoor Tanning and Sociocultural Influences

A paradoxical behavior observed among young women is the persistent popularity of indoor tanning, despite widespread awareness of its carcinogenic potential. Indoor tanning is a well-established risk factor for both melanoma and non-melanoma skin cancers, particularly in women under the age of 25 [15]. Many users acknowledge the associated cancer risk but continue to tan due to sociocultural and psychological motivations. Studies indicate that young women often prioritize short-term, appearance-related outcomes—such as improved self-esteem or perceived attractiveness—over long-term health considerations [16]. The enduring perception of tanned skin as a symbol of health, beauty, or social status further reinforces this behavior [17].

Women of reproductive age demonstrate relatively high awareness of melanoma prevention, yet substantial behavioral gaps and disparities persist. Gender norms, cultural influences, and misconceptions continue to shape risk behaviors such as tanning and inadequate photoprotection. Targeted public health interventions—especially those addressing ethnic disparities and appearance-based motivations—are essential to improving melanoma prevention and early detection in this population.

## 3. Preventive Behaviors and Photoprotection Strategies

Preventive behaviors play a crucial role in reducing the risk of skin cancer, particularly in vulnerable populations such as pregnant women. During pregnancy, hormonal and immunological changes may influence skin sensitivity and potentially alter the appearance of nevi, underscoring the importance of consistent sun protection practices [18]. A comprehensive photoprotection approach should involve wearing wide-brimmed hats, clothing designed to block the sun’s rays, and consistently applying high-SPF. These broad-spectrum sunscreens offer protection against UVB, UVA, and high-energy visible light [19]. While sunscreen use is generally considered safe, it is essential to choose products with minimal potential exposure to harmful chemicals, especially during the first trimester and breastfeeding. UV filters should be used by pregnant women just as much as by non-pregnant women. For adequate sun protection, the WHO recommends using a broad-spectrum sunscreen with SPF 30 or higher that blocks both UVA and UVB rays and is water resistant for 4 h [20].

During pregnancy, caution is advised regarding certain cosmetic ingredients because of their potential effects on the developing fetus. Sunscreen use is generally considered safe during pregnancy, provided that appropriate formulations are selected. In clinical practice, mineral (inorganic) filters, such as zinc oxide and titanium dioxide, are preferred because they are minimally absorbed through the skin and lack known endocrine activity. These products are regarded as the safest options for both pregnant and breastfeeding women. Their main disadvantages—heavier texture and a whitish appearance on the skin—can be mitigated by using hybrid formulations that combine mineral filters with selected low-absorption organic filters that have demonstrated good safety profiles [19,20,21,22]. Although current evidence supports the safety of these approaches, data on breastfeeding women remain limited, and further studies are warranted to confirm long-term safety.

Effective photoprotection includes not only the use of UV filter-containing products, but also several non-pharmacological strategies that play a significant role in limiting exposure to harmful UV radiation and visible light. The most basic prevention method is avoiding sun exposure, especially during peak sunlight hours (between 10:00 a.m. and 4:00 p.m.). UVA penetrates window glass, so people spending long periods near windows should consider additional protection. For blue light (HEV), clinically meaningful pigmentary effects are better documented in darker Fitzpatrick phototypes; in lighter phototypes, the impact is less clear [23]. Seeking shade is a practical and accessible solution, although it is important to note that UV radiation can still reach the skin after reflecting off horizontal surfaces. This reduces the effectiveness of this method unless it is combined with other protective measures. Additional methods include physical protective barriers, such as clothing—especially wide-brimmed hats, which shield the head, face, and neck — as well as umbrellas and sunshades, whose effectiveness depends on the material and UV permeability [24,25]. Sunglasses are another essential element of sun protection. Their effectiveness depends on lens geometry, how they are worn, how well they fit the face, and the quality of the UV filters used. The best protection is provided by goggles that fit snugly, limiting light entry from different angles. The importance of selecting protective clothing properly is also emphasized. Fabrics with a high ultraviolet protection factor, especially densely woven and dark synthetic fabrics, serve as a significant barrier, reducing UV transmission through clothing. Modern textiles may also be enriched with UV-absorbing substances or coated with light-reflecting materials [23].

Regular self-examination of skin lesions and dermatological check-ups should also be emphasized. There are no guidelines suggesting that the frequency of skin exams should change during pregnancy; therefore, it is assumed that pregnant women should undergo check-ups at the same frequency as non-pregnant individuals. Routine screening should be performed annually. Exceptions include individuals with: a personal or family history of melanoma, numerous pigmented nevi, or atypical mole syndrome (for whom monitoring is recommended every 3–6 months) [26]. Dermatologists should rely on standard clinical and dermoscopic guidelines when assessing suspicious skin lesions in pregnant patients [27]. To facilitate patient self-examination of moles, several mnemonic methods have been developed to help recognize concerning features. One of the most well-known is the ABCDE rule—Asymmetry, Border irregularity, Color variation, Diameter > 6 mm, and Evolution —remains the cornerstone of melanoma risk stratification and must be applied rigorously in pregnant patients [28,29]. However, recent studies have demonstrated that a proportion of invasive melanomas present with diameters smaller than 6 mm, underscoring that the “D” parameter should not be interpreted as an absolute threshold but rather as a relative indicator of risk. Accordingly, any new, evolving, or atypical pigmented lesion, regardless of size, warrants prompt dermoscopic evaluation and, when clinically justified, biopsy [30,31]. 

Another important aspect of skin cancer prevention in pregnant women is patient education. It is worth noting that, during prenatal care, most women do not receive advice or information regarding the benefits of sun protection or the warning signs of skin lesions [32]. Increased access to information, public health campaigns, and the inclusion of these recommendations during routine gynecological visits may significantly enhance patient awareness of the importance of using various photoprotective methods during pregnancy and the postpartum period.

## 4. Pregnancy-Associated Changes in Melanocytic Nevi

Melanocytic nevi are benign proliferations of melanocytes, histologically defined by nests or aggregations of melanocytes within the epidermis, dermis, or both, and clinically manifesting as variably pigmented macules, papules, or nodules [33]. These lesions are classified into subtypes, including junctional, compound, intradermal, blue, and Spitz nevi, based on their anatomical distribution, histopathological architecture, and clinical morphology [34]. Nevi can be congenital or acquired later in life. Congenital melanocytic nevi arise from somatic mutations during embryogenesis and are typically present at birth or in early infancy [28]. In contrast, acquired nevi develop later in life, predominantly during childhood and adolescence, and their number and morphology are influenced by genetic factors and cumulative UV exposure [33]. At the molecular level, nevogenesis is driven by activating mutations in the MAPK signaling pathway, most notably in BRAF rand NRAS, with specific mutations associated with distinct nevus phenotypes and anatomical sites [34].

Pregnancy induces substantial hormonal, immunological, and metabolic changes, including increased levels of estrogen, progesterone, and melanocyte-stimulating hormone, which affect melanogenesis and cutaneous pigmentation [29,35]. These physiological alterations commonly provoke clinical concern about potential changes in melanocytic nevi during pregnancy, particularly morphological changes that may complicate the differentiation between benign nevus change and melanoma [36].

Most available data derive from small single-center prospective or observational cohorts. Consequently, conclusions regarding nevus behavior during pregnancy should be interpreted with caution. Current ESMO (2023) [37] and AAD (2022) [38] melanoma guidelines emphasize clinical vigilance but do not recommend altering standard surveillance intervals [37,38].

Larger multicenter studies and standardized imaging protocols, including long-term postpartum follow-up, are needed to better distinguish physiological changes from early signs of malignancy.

## 5. Hormonal and Immunological Influences on Melanocytic Nevi During Pregnancy

Pregnancy is characterized by significant hormonal and immunological changes that can affect melanocytic nevi. Estrogen and progesterone levels increase progressively throughout gestation, promoting melanogenesis through pathways mediated primarily by estrogen receptor β (ERβ), the predominant receptor subtype expressed in melanocytic nevi [39,40]. Immunohistochemical studies demonstrate significantly elevated ERβ expression in nevi during pregnancy, especially in epidermal and superficial dermal nevocytes, indicating enhanced hormonal responsiveness [39].

Despite these molecular changes, most prospective studies do not confirm consistent clinical or structural alterations in nevi during pregnancy. Spectrophotometric analysis reveals no significant modifications in size, pigmentation, or vascularity between nevi of pregnant and nonpregnant women [41]. Observed increases in nevus size are frequently noted on the abdomen or breasts, regions subject to mechanical stretching, and are likely attributable to cutaneous distension rather than true melanocytic proliferation [42].

Progesterone may modulate melanocyte function through non-classical receptors such as PAQR7, which has been shown to suppress melanin synthesis and promote autophagy; however, its overall role remains incompletely understood [43]. Additionally, pregnancy induces immunologic tolerance by shifting toward a Th2-dominant cytokine profile and upregulating immune-modulatory factors, such as indoleamine 2,3-dioxygenase, which may influence nevus surveillance and behavior [44].

Although hormonal and immunologic alterations during pregnancy affect melanocyte biology at the molecular level, current evidence does not support any clinically meaningful changes in the morphology or behavior of melanocytic nevi. The strength of evidence is limited by small sample sizes, heterogeneous and variable follow-up, which constrain robust inference. Where stated, practical recommendations reflect expert consensus rather than high-level evidence. Prospective multicenter studies with standardized imaging and longitudinal postpartum follow-up are needed to clarify the clinical translation of these biological shifts.

## 6. Clinical and Dermoscopic Changes in Nevi

Several prospective studies report that the most common clinical changes in nevi during pregnancy include size enlargement and alterations in pigmentation, primarily on the abdomen and breasts, areas subject to mechanical stretching during gestation [35]. Most have consistently shown that changes, when present, are subtle, localized, and predominantly physiological. The most commonly reported findings include slight enlargement and pigmentary variation in nevi located on the abdomen and breasts, areas subject to skin stretching and mechanical distension, while lesions on the back and extremities remain largely unchanged [45,46,47,48].

In a prospective cohort study of 18 pregnant women (mean age 33 years), total body photography and digital dermoscopy of 703 nevi revealed that 55% of nevi enlarged in size, most frequently on the abdomen (87.1%, *p* < 0.001), and 44% of patients developed new melanocytic lesions, primarily on the upper limbs [45]. Similarly, Strumia et al. found that reticular-pattern nevi on the abdomen and breasts became more widely meshed and lighter, while globular nevi developed peripheral brown globules. These changes were largely confined to mechanically stretched regions [46].

By contrast, studies assessing nevi on relatively non-distensible areas, such as the back or lower limbs, reported no clinically meaningful enlargement during pregnancy. In a prospective survey by Pennoyer et al., 129 back nevi in 22 pregnant women were monitored between the first and third trimesters of pregnancy. Only 6.2% demonstrated any change in diameter, with an equal distribution between increases and decreases, and a mean change of 0 mm. This suggests that local mechanical skin stretching, rather than systemic hormonal modulation, primarily influences nevus morphology in these regions [47].

Supporting this view, Wyon et al. conducted a spectrophotometric study of 381 nevi in 34 primigravidae and 163 nevi in 21 nonpregnant controls using SIAscopy. Focusing on lesions located on the back and lower legs, they found no statistically significant changes in size, pigmentation, or dermoscopic architecture. Minor variations, such as a 2.1% increase in size and a 3.7% decrease in pigment, were comparable to controls and not considered biologically meaningful. The authors concluded that pregnancy does not significantly affect nevi in non-distensible regions [41].

However, mechanical factors alone may not fully account for the observed changes. Several studies report alterations in nevi located on non-distensible areas, which may suggest hormonal involvement. In a prospective AI-assisted dermoscopic study involving 2799 nevi, Peter et al. documented significant increases in diameter, area, color diversity, and shape asymmetry from the first trimester to the postpartum period, including changes on non-distensible sites (back and extremities) [48]. Similarly, Zampino et al. evaluated 86 nevi located on the backs of 47 pregnant women and reported no significant changes in size; however, they observed a marked increase in dermoscopic vascular structures and total dermoscopy score (TDS) during the third trimester, both of which regressed postpartum. These results support the idea of reversible hormonal influences on melanocytic activity [49].

In a study by Aktürk et al., 97 nevi in 56 pregnant women were examined between the first and third trimesters of pregnancy. A statistically significant increase in size and TDS was observed, with new dot formation in six lesions. These changes were not confined to stretched skin, reinforcing the role of intrinsic, pregnancy-associated biological modulation [50].

Patients with dysplastic nevus syndrome (DNS) may demonstrate heightened sensitivity to gestational changes. A longitudinal study of 17 women with DNS followed across 22 pregnancies found that 76% exhibited clinical changes in nevi, with a 3.9-fold higher rate of change compared to the nonpregnant state. Biopsied lesions during pregnancy were twice as likely to display histologic dysplasia [51].

Dermoscopic evaluation is essential in documenting pregnancy-related changes. A review by Cosgarea et al. of six cohort studies involving 1167 nevi in 258 pregnant women identified consistent dermoscopic alterations, including increased peripheral globules, dot formation, new vascular structures, and remodeling of the pigment network. Importantly, no cases of melanoma were reported, reinforcing the benign and physiological nature of these changes [52].

Rubegni et al. analyzed 206 nevi from 35 pregnant and 35 nonpregnant women, reporting pigment network thickening and globule darkening during pregnancy, with pigmentation generally returning to baseline 12 months postpartum. Interestingly, some architectural changes persisted, suggesting that not all modifications are transient [53].

The overall strength of evidence is moderate to low. Most available studies are small, single-center prospective cohorts, using heterogeneous dermoscopic or imaging endpoints and limited postpartum follow-up. Despite these methodological constraints, their consistent results support a predominantly benign and reversible pattern of nevus changes during pregnancy. Clinicians should, however, remain vigilant, as physiological alterations may occasionally mimic early melanoma. Conclusions should therefore be interpreted cautiously and framed within the context of these limitations.

Table 1 summarizes prospective studies assessing changes in melanocytic nevi during pregnancy, including details on study design, number of participants and nevi analyzed, anatomical site of lesions, main clinical observations, observation period during pregnancy, and post-pregnancy follow-up.

## 7. Diagnostic and Management Considerations During Pregnancy

Changes in melanocytic nevi during pregnancy, particularly in size, shape, or pigmentation, require careful diagnostic evaluation and should not be presumed benign or hormonally mediated without objective assessment [35]. Dermoscopic modifications, such as thickening of the pigment network, increased globules, and elevated total dermoscopy scores, are commonly observed in nevi on the abdomen and breasts during pregnancy. Although these typically resolve postpartum and do not independently necessitate intervention, rapidly evolving or equivocal lesions warrant histopathologic evaluation, particularly when nodular or heavily pigmented features raise concern for melanoma [54,55].

Significantly, clinical decisions should not be delayed when suspicious features are present, even in the absence of concerning dermoscopic criteria [30]. The classic ABCDE criteria, which outlines warning signs. Any change in size, shape, color, structure, or the appearance of symptoms like itching, bleeding, or irritation [56]. However, it is important to remember to watch out for alarming signs even in the case of lesions smaller than 6 mm which are even up to 38% of cases [31].

These criteria retain their diagnostic validity and should prompt immediate dermatologic referral and consideration for biopsy when present [29].

Biopsy of concerning nevi during pregnancy is considered safe and should not be deferred. Excisional biopsy under local anesthesia (lidocaine, FDA Category B), with or without low-dose epinephrine (1:200,000), is safe in all trimesters. However, the second trimester is preferred when possible to minimize procedural and anesthetic risk [54]. Furthermore, histologic interpretation must account for gestational status, as pregnancy-associated features, such as increased mitotic activity, superficial micronodules, or mildly elevated Ki-67 index, may mimic atypia without signifying malignancy [35,36]. Therefore, clinicians should clearly indicate pregnancy status on pathology requisition forms to facilitate accurate clinicopathologic correlation [55].

When melanoma is suspected or confirmed, prompt wide local excision should be performed in accordance with standard guidelines. Sentinel lymph--node biopsy (SLNB) may be considered for invasive melanomas with conventional indications (e.g., ≥1.0 mm Breslow). Current ESMO (2023) [37] and AAD (2019) [38] guidelines specify that SLNB is not routinely recommended for pT1a melanomas but may be discussed in selected cases with high-risk features (e.g., ≥3 mitoses/mm^2^, ulceration, lymphovascular invasion, a positive deep margin, or uncertain Breslow thickness). It should be offered to patients with pT1b melanoma (>0.8–1.0 mm or <0.8 mm with ulceration) and is recommended for all clinically node-negative stage T2a or higher tumours. Although SLNB confers no direct therapeutic benefit, it provides critical prognostic and staging information that guides eligibility for adjuvant systemic or targeted therapy. For thick (T3b–T4b) melanomas where adjuvant treatment is already indicated, omission of SLNB may be considered after multidisciplinary discussion [37,38].

In pregnancy, SLNB can be performed with technetium-99m sulfur colloid alone (low fetal radiation exposure; individualized decision). Isosulfan blue is generally avoided because of the risk of anaphylaxis. Methylene blue should be avoided during the first trimester due to its association with fetal intestinal atresia and is commonly avoided throughout pregnancy [27,36]. For staging purposes, ultrasound and non-contrast MRI are preferred; chest radiography with abdominal shielding is permissible, while CT and PET should be reserved for situations in which the diagnostic benefits outweigh the risk of fetal radiation [27,36]. According to the ESMO (2023) [37] Clinical Practice Guidelines, no additional imaging is required for low-risk primary cutaneous melanomas (pT1a). In higher tumour stages, ultrasound of regional lymph nodes and/or CT or PET imaging may be used to evaluate tumour extension and guide the indication for sentinel lymph-node biopsy (SLNB). For patients with disease stage IIB or higher, brain MRI is recommended to complete staging. These risk-stratified recommendations support individualized timing of imaging in pregnant patients, balancing maternal benefit with fetal safety [37].

Most data informing diagnostic and management recommendations are derived from retrospective series, small cohorts, and expert consensus, as randomized studies are ethically unfeasible in pregnant populations. Despite these limitations, the described approach is consistent with international melanoma guidelines (ESMO 2023; AAD 2022 [37,38]) and represents current best practice. Clinicians should interpret available evidence cautiously and maintain a high level of vigilance when assessing changing nevi during pregnancy.

In conclusion, pregnancy should not alter the standard of care for melanocytic nevi: clinical vigilance, timely biopsy, and interdisciplinary management remain the cornerstones of safe and effective care.

## 8. Systemic Treatment: BRAF Inhibitors and Immunotherapy

### 8.1. BRAF Inhibitors

In pregnant patients with advanced-stage melanoma, treatment data remain particularly scarce. Targeted therapies with BRAF (vemurafenib, dabrafenib, encorafenib) and MEK inhibitors (cobimetinib, trametinib, binimetinib) are used in the treatment of patients with BRAF-mutant melanoma [57]. Preclinical studies in murine models have demonstrated potential fetal toxicity associated with several targeted agents. For instance, dabrafenib and MEK inhibitors have shown teratogenic and embryotoxic effects in animal (pup and mouse) studies [44,58,59]. According to the U.S. Food and Drug Administration (FDA), both BRAF and MEK inhibitors are classified as pregnancy category D, indicating evidence of fetal risk. Human pregnancy data consist primarily of isolated case reports with vemurafenib, showing variable maternal responses and generally reassuring neonatal outcomes, but also severe maternal adverse events (e.g., toxic epidermal necrolysis) in at least one case [60,61,62,63] (Table 2).

A case, reported by Maleka et al. [60], involved a woman diagnosed with metastatic melanoma at 25 weeks of gestation. She was treated with vemurafenib (960 mg twice daily), resulting in a notable reduction in metastatic disease. Despite pre-existing fetal growth restriction, the pregnancy continued until 30 weeks, when a cesarean section was performed due to fetal distress. The neonate was born without congenital anomalies. The patient survived the pregnancy but succumbed to disease progression approximately 2.5 months postpartum [60].

De Haan et al. [61] described a 30-year-old woman with a monochorionic–diamniotic twin pregnancy. Vemurafenib (twice a day, 960 mg) was initiated at 22 weeks’ gestation. After 12 days of treatment, the patient developed severe toxic epidermal necrolysis, a life-threatening dermatologic adverse effect. Emergency vaginal delivery of both twins occurred at 26 weeks while the patient was sedated in the intensive care unit. Both neonates survived without structural abnormalities. However, the patient died several weeks later due to an intracranial hemorrhage related to metastatic disease [61].

Another case reported by Marcé et al. [62] described a favorable maternal response to vemurafenib (480 mg twice daily) initiated during pregnancy. No adverse maternal or fetal effects were reported. The pregnancy continued without complication, and the neonate was delivered without evidence of congenital or developmental abnormalities [62].

Pagan et al. [63] described a 25-year-old pregnant woman diagnosed with metastatic melanoma at 20 weeks of gestation. After declining pregnancy termination, she began treatment with vemurafenib at 25 weeks, following multidisciplinary counseling. The therapy was well tolerated, and fetal development was closely monitored. At 34 weeks, labor was induced to allow further oncologic treatment. A healthy infant was delivered vaginally, with no congenital anomalies. The neonatal course was complicated by supraventricular tachycardia, managed successfully with beta-blockers. The mother had an uncomplicated pregnancy and delivery [63].

Taken together, these cases suggest that vemurafenib may induce a favorable antitumor response in pregnant patients with BRAF V600-mutant melanoma. Fetal outcomes have been generally reassuring, with no reported teratogenic effects directly attributed to the drug. However, maternal safety remains a significant concern, particularly in light of the severe cutaneous toxicity observed in one case. Due to the limited evidence base and potential for life-threatening adverse events, the decision to initiate vemurafenib during pregnancy should be made on an individual basis by a multidisciplinary team, carefully balancing maternal prognosis with potential fetal risks.

### 8.2. Immunotherapy

The primary immunotherapeutic agents approved for the treatment of metastatic melanoma include the monoclonal antibodies nivolumab, pembrolizumab, and ipilimumab. Nivolumab and pembrolizumab inhibit the programmed cell death-1 (PD-1) receptor on T cells, whereas ipilimumab targets cytotoxic T-lymphocyte-associated antigen 4 (CTLA-4) [64].

CTLA-4 and PD-1 are involved in maintaining maternal immune tolerance toward the fetus [65]. In reproductive studies conducted on monkeys, treatment with ipilimumab, nivolumab, and pembrolizumab was associated with dose-dependent increases in abortion, stillbirth, preterm delivery, and elevated infant mortality. These adverse effects are presumed to be more pronounced during treatment in the second and third trimesters of gestation [66,67].

In the general treatment paradigm for advanced melanoma, the phase III DREAMSeq trial compared first-line combination immunotherapy with nivolumab and ipilimumab versus targeted therapy with dabrafenib and trametinib in patients with BRAF-mutated metastatic disease. The study demonstrated significantly higher 2-year overall survival (72% vs. 52%) and longer progression-free survival among patients treated first with immunotherapy, establishing it as the preferred initial treatment approach in non-pregnant individuals. In pregnancy, however, immune checkpoint inhibitors should be reserved for life-threatening maternal disease and used only after multidisciplinary risk assessment.

A systematic review of case reports and pharmacovigilance data (2000–2021) identified seven pregnancies exposed to immune checkpoint inhibitors (ICIs), mainly in patients with metastatic melanoma. Immunotherapeutic approaches primarily involved nivolumab, either as monotherapy or combined with ipilimumab, while ipilimumab alone was used in only one reported case. ICIs were administered for an average of 9.8 weeks, with most births occurring preterm (mean gestational age: 30.4 weeks; mean birth weight: 1267 g). Obstetric complications were common, including intrauterine growth restriction and placental disorders. While maternal outcomes showed some clinical benefit, fetal risks were significant, underscoring the need for cautious, multidisciplinary management [68]. The use of pembrolizumab as monotherapy has also been reported. Anami et al. published a case of a 40-year-old primigravida with advanced melanoma diagnosed in the first trimester, who continued the pregnancy and received pembrolizumab between weeks 21 and 27. Delivery was carried out via cesarean section at 28 weeks, resulting in the birth of a clinically stable neonate. Starting on postpartum day 13, the patient was treated with a combination of nivolumab and ipilimumab, followed by maintenance therapy with nivolumab alone. The disease has remained controlled, and the patient has survived for 20 months to date [69]. Current clinical guidelines typically advise against the routine use of immunotherapy in pregnant patients unless the potential benefit significantly outweighs the risks. A summary of the studies discussed in this subsection is presented in Table 3.

Other immunotherapies, including talimogene laherparepvec (T-VEC) and interleukin-2 (Proleukin), are established treatment options for melanoma in the general population. However, their safety and efficacy in pregnant women have not been adequately studied. To date, no clinical studies have evaluated the safety or efficacy of T-VEC in pregnant women. Nevertheless, preclinical investigations in pregnant murine models have not demonstrated any adverse effects on embryo-fetal development [70]. Interleukin-2 demonstrated embryolethal effects in rats at doses substantially higher than those used in humans. Maternal toxicity was observed at moderately to markedly elevated doses during organogenesis [71]. Both T-VEC and interleukin-2 are generally contraindicated during pregnancy due to the absence of data on fetal safety and the potential risk of maternal and embryofetal toxicity.

**Figure 1:**
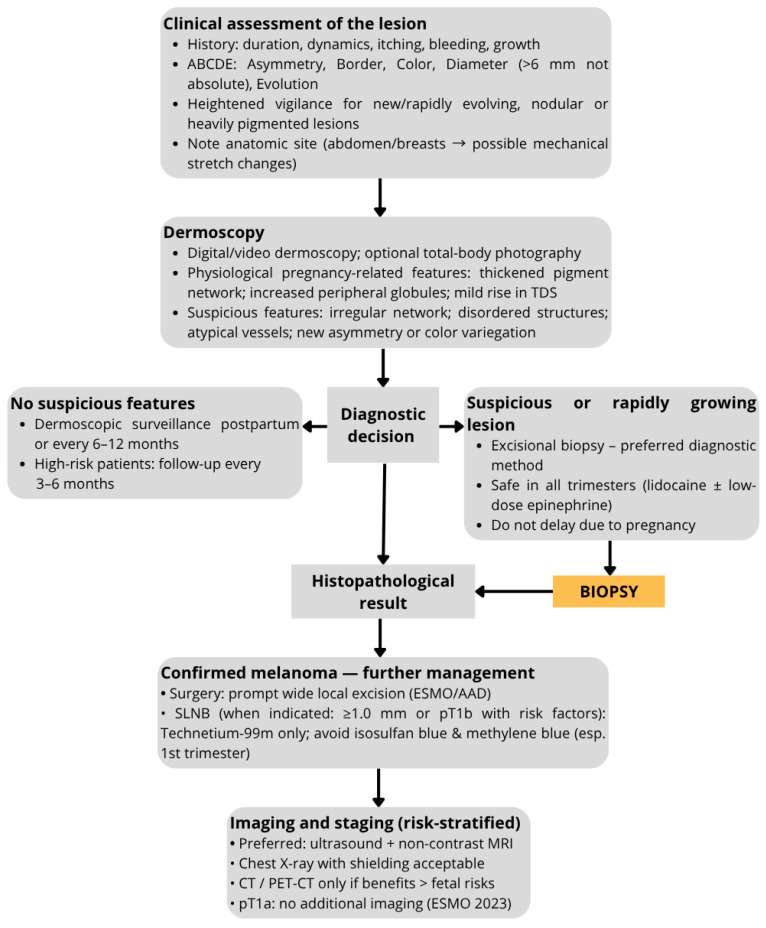
Diagnostic and management algorithm for melanocytic lesions during pregnancy. The flowchart outlines a stepwise approach, beginning with a clinical assessment of the lesion and dermoscopic evaluation. Based on diagnostic decision-making, lesions without suspicious features are monitored through dermoscopic surveillance, whereas suspicious or rapidly growing lesions undergo biopsy. Histopathological results guide further management: confirmed melanoma requires surgical excision, possible sentinel lymph node biopsy (SLNB), and imaging/staging as risk-stratified [37,38].

Figure 1 presents the proposed diagnostic and management algorithm for melanocytic lesions during pregnancy.

## 9. Conclusions

The rising incidence of skin cancer among women of reproductive age underscores the need for targeted prevention strategies and education. Hormonal and immunological changes during pregnancy can alter the appearance of melanocytic nevi, emphasizing the importance of regular skin monitoring throughout this period. While current guidelines for skin examinations remain the same during pregnancy, incorporating skin cancer education into routine prenatal care could enhance early detection and awareness. While current diagnostic thresholds and surgical standards do not change due to pregnancy, effective communication and coordination among dermatology, obstetrics, oncology, radiology, and pediatrics are essential. Public health efforts should prioritize equitable awareness initiatives that reach populations with a lower perceived risk, pragmatic, pregnancy-appropriate photoprotection (favoring mineral sunscreens and sun-avoidance behaviors), streamlined pathways for prompt evaluation and biopsy of suspicious lesions, and clear, risk-based follow-up schedules. Advanced melanoma in pregnancy remains a major therapeutic challenge; decisions about systemic therapy must be individualized, acknowledge the paucity of evidence, and center on maternal survival while minimizing fetal risk.

Pregnancy is a unique condition in which therapeutic decisions for melanoma may be delayed or limited due to ethical, medical, and social factors. This can lead to inequities in access to optimal care compared with non-pregnant patients. To address these disparities, international registries and multicenter cohorts (e.g., a Melanoma Pregnancy Registry) are needed. Such initiatives would improve data collection, inform evidence-based management, and promote gender equity in melanoma treatment during pregnancy.

## Figures and Tables

**Table 1:** Overview of prospective studies evaluating changes in melanocytic nevi during pregnancy.

Study	Participants/Number of Analyzed Nevi	Anatomical Site	Main Observations	Period	Follow-Up
Wyon et al. [41]	34 pregnant, 21 controls/544 nevi	Back and lower legs	No significant change in size or pigmentation; minor variation similar to controls	1st trimester to 37th week	Not assessed
Martins-Costa & Bakos [45]	18 women/703 nevi	abdomen, upper limbs	55% enlarged, mostly abdominal; increased or decreased pigmentation; network changes; appearance of new dots and globules; emergence of vascular structures, streaks, and structureless areas.	1st to 3rd trimester	Not assessed
Strumia et al. [46]	12 women/56 nevi	Whole body	Pigment network clearer and more widely meshed with growth; more brown globules at the periphery with growth.	2nd to 3rd trimester	Not assessed
Pennoyer et al. [47]	22 women/129 nevi	Back	6.2% diameter change; mean size unchanged.	1st to 3rd trimester	None
Peter et al. [49]	25 pregnant, 25 controls/2799 nevi	Whole body, including back, limbs	Increased size, asymmetry, and color diversity across all regions.	1st trimester to postpartum	Immediate postpartum
Zampino et al. [48]	47 women/86 nevi	Back	Increased vascular structures and TDS; no size change.	1st to 3rd trimester	6 months postpartum
Aktürk et al. [50]	56 women/97 nevi	Whole body	Increased TDS; new dot formation.	1st to 3rd trimester	Not assessed
Rubegni et al. [53]	35 pregnant, 35 controls/206 nevi	Excluded breasts, abdomen, and acral	Increased thickness and prominence of the pigment network; darker globules in nevi with a globular pattern; reduced structural coherence of the reticular pattern; decreased homogeneity in globule size and distribution.	During pregnancy	12 months postpartum

**Table 2:** Summary of vemurafenib use in pregnant patients with metastatic melanoma.

References and Year of Publication	Maternal Age	Gestational Age at Treatment	Pregnancy Type	Delivery	Neonatal Outcome	Maternal Outcome
Maleka et al., 2013 [60]	37 years	25 weeks	Singleton	Cesarean at 30 weeks	Healthy,	3-month progression-free survival,
de Haan et al., 2018 [61]	30 years	22 weeks	Twin	Preterm delivery at 26 weeks	Both infants healthy,	Severe toxic epidermal necrolysis, 53 days after delivery and 78 days after the start of vemurafenib, died of an intracranial haemorrhage due to metastatic disease.
Marcé et al., 2019 [62]	29 years	17 weeks	Singleton	Delivery at 36 weeks	Healthy,	Partial response,
Pagan et al, 2019 [63]	25 years	25 weeks	Singleton	Delivery at 34 weeks	Healthy infant. The neonatal course was complicated by paroxysmal supraventricular tachycardia.	Reduction in the size of pulmonary and cutaneous metastases,

**Table 3:** Summary of studies on the use of immune checkpoint inhibitors (ICIs) during pregnancy in patients with metastatic melanoma.

Reference	Immunotherapeutic Agent(s)	Gestational Timing of Exposure	Duration of Treatment	Delivery	Fetal/Neonatal Outcome	Maternal Outcome
Andrikopoulou et al. (Systematic review, 2000–2021) [68]	Nivolumab ± Ipilimumab; Ipilimumab alone (1 case)	Mostly 2nd–3rd trimesters	Mean: 9.8 weeks	High rate of preterm delivery (mean GA: 30.4 weeks)	Mean birth weight: 1267 g; intrauterine growth restriction and placental disorders common; elevated infant mortality	Some maternal clinical benefit; requires multidisciplinary management
Anami et al. (Case report) [69]	Pembrolizumab (monotherapy, then Nivolumab + Ipilimumab postpartum)	Pembrolizumab between 21 and 27 weeks; postpartum combination therapy	6 weeks	Cesarean section at 28 weeks	Clinically stable neonate; preterm	Disease controlled; 20 months survival to date

## Data Availability

Data sharing is not applicable to this article as no datasets were generated or analyzed.

## Abbreviations

The following abbreviations are used in this manuscript:

PAM	pregnancy-associated melanoma
UV	ultraviolet
EDC	endocrine-disrupting chemical
Erβ	estrogen receptor β
TDS	total dermoscopy score
DNS	dysplastic nevus syndrome
PD-1	programmed cell death-1
CTLA-4	cytotoxic T-lymphocyte-associated antigen 4
ICIs	immune checkpoint inhibitors
T-VEC	talimogene laherparepvec

## References

[1] Simões M.C.F., Sousa J.J.S., Pais A.A.C.C. (2015). Skin Cancer and New Treatment Perspectives: A Review. Cancer Lett..

[2] Lens M., Bataille V. (2008). Melanoma in Relation to Reproductive and Hormonal Factors in Women: Current Review on Controversial Issues. Cancer Causes Control.

[3] Byrom L., Olsen C., Knight L., Khosrotehrani K., Green A.C. (2015). Increased Mortality for Pregnancy-Associated Melanoma: Systematic Review and Meta-Analysis. J. Eur. Acad. Dermatol. Venereol..

[4] Kyrgidis A., Lallas A., Moscarella E., Longo C., Alfano R., Argenziano G. (2017). Does Pregnancy Influence Melanoma Prognosis? A Meta-Analysis. Melanoma Res..

[5] Lambe M., Thörn M., Sparén P., Bergström R., Adami H.O. (1996). Malignant Melanoma: Reduced Risk Associated with Early Childbearing and Multiparity. Melanoma Res..

[6] Kreuz M., de Souza Wagner P.H., Tanimoto L.E., da Rosa V.A., Talah B.A.D., de Moraes F.C.A. (2025). Melanoma and Pregnant Women: A Systematic Review and Meta-Analysis. Melanoma Res..

[7] Bradford P.T., Anderson W.F., Purdue M.P., Goldstein A.M., Tucker M.A. (2010). Rising Melanoma Incidence Rates of the Trunk among Younger Women in the United States. Cancer Epidemiol. Biomark. Prev..

[8] Łyko M., Kruzel M., Kuś A., Maj J., Szepietowski J.C., Jankowska-Konsur A. (2021). Sun Protection among University Students in Poland: A Survey of Awareness and Attitudes. Postep. Dermatol. Alergol..

[9] Koumaki D., Evangelou G., Gregoriou S., Kouloumvakou S., Manios A., Katoulis A., Zacharopoulos G.V., Chernyshov P.V., Papadakis M., Kassotakis D. (2024). Skin Cancer Knowledge, Sun Exposure, Photoprotection Behavior, and Perceived Barriers Associated with Skin Cancer Types in a Greek Cohort: A Cross-Sectional Study on the Island of Crete. Cancers.

[10] Woźna J., Stępka J., Bałoniak A., Adamski Z. (2024). Evaluation of Social Knowledge on Photoprotection and Its Relationship with Education and Age in a Polish Seaside Town during Summer Holidays. Photodermatol. Photoimmunol. Photomed..

[11] Lee A., Garbutcheon-Singh K.B., Dixit S., Brown P., Smith S.D. (2015). The Influence of Age and Gender in Knowledge, Behaviors and Attitudes towards Sun Protection: A Cross-Sectional Survey of Australian Outpatient Clinic Attendees. Am. J. Clin. Dermatol..

[12] Al-Dujaili Z., Henry M., Dorizas A.S., Sadick N.S. (2017). Skin Cancer Concerns Particular to Women. Int. J. Womens Dermatol..

[13] Schwartz M.R., Luo L., Berwick M. (2019). Sex Differences in Melanoma. Curr. Epidemiol. Rep..

[14] Buchanan Lunsford N., Berktold J., Holman D.M., Stein K., Prempeh A., Yerkes A. (2018). Skin Cancer Knowledge, Awareness, Beliefs and Preventive Behaviors among Black and Hispanic Men and Women. Prev. Med. Rep..

[15] Wehner M.R., Shive M.L., Chren M.M., Han J., Qureshi A.A., Linos E. (2012). Indoor Tanning and Non-Melanoma Skin Cancer: Systematic Review and Meta-Analysis. BMJ.

[16] Newton J., Ochoa L., Reinschmidt A., Vassar J., Wellman A., Vargas M., Kenyon D., Frohm M. (2024). Is Beauty Worth the Risk? Self-Confidence Is the Key Motivating Factor Driving Tanning Bed Use among Undergraduate Students at South Dakota Universities. Int. J. Womens Dermatol..

[17] Lyons S., Lorigan P., Green A.C., Ferguson A., Epton T. (2021). Reasons for Indoor Tanning Use and the Acceptability of Alternatives: A Qualitative Study. Soc. Sci. Med..

[18] Gupta S.N., Madke B., Ganjre S., Jawade S., Kondalkar A. (2024). Cutaneous Changes During Pregnancy: A Comprehensive Review. Cureus.

[19] Lim H.W., Piquero-Casals J., Schalka S., Leone G., Trullàs C., Brown A., Foyaca M., Gilaberte Y., Krutmann J., Passeron T. (2025). Photoprotection in Pregnancy: Addressing Safety Concerns and Optimizing Skin Health. Front. Med..

[20] Radiation: Protecting Against Skin Cancer. https://www.who.int/news-room/questions-and-answers/item/radiation-protecting-against-skin-cancer.

[21] Biskanaki F., Tertipi N., Andreou E., Sfyri E., Kefala V., Rallis E. (2024). The Risk of Using Cosmetics and Cosmetic Procedures During Pregnancy. Appl. Sci..

[22] Matta M.K., Zusterzeel R., Pilli N.R., Patel V., Volpe D.A., Florian J., Oh L., Bashaw E., Zineh I., Sanabria C. (2019). Effect of Sunscreen Application Under Maximal Use Conditions on Plasma Concentration of Sunscreen Active Ingredients: A Randomized Clinical Trial. JAMA.

[23] Zhao L., Fu X., Cheng H. (2024). Prevention of Melasma During Pregnancy: Risk Factors and Photoprotection-Focused Strategies. Clin. Cosmet. Investig. Dermatol..

[24] Glaser K.S., Tomecki K.J. (2020). Sunscreens in the United States: Current Status and Future Outlook. Adv. Exp. Med. Biol..

[25] McDonald K.A., Lytvyn Y., Mufti A., Chan A.W., Rosen C.F. (2023). Review on Photoprotection: A Clinician’s Guide to the Ingredients, Characteristics, Adverse Effects, and Disease-Specific Benefits of Chemical and Physical Sunscreen Compounds. Arch. Dermatol. Res..

[26] Friedman R.J., Farber M.J., Warycha M.A., Papathasis N., Miller M.K., Heilman E.R. (2009). The “Dysplastic” Nevus. Clin. Dermatol..

[27] Berk-Krauss J., Liebman T.N., Stein J.A. (2018). Pregnancy and Melanoma: Recommendations for Clinical Scenarios. Int. J. Womens Dermatol..

[28] Frischhut N., Zelger B., Andre F., Zelger B.G. (2022). The Spectrum of Melanocytic Nevi and Their Clinical Implications. J. Dtsch. Dermatol. Ges..

[29] Bieber A.K., Martires K.J., Stein J.A., Grant-Kels J.M., Driscoll M.S., Pomeranz M.K. (2017). Pigmentation and Pregnancy: Knowing What Is Normal. Obstet. Gynecol..

[30] Slowinska M., Kaminska-Winciorek G., Kowalska-Oledzka E., Czarnecka I., Czarnecki R., Nasierowska-Guttmejer A., Paluchowska E., Owczarek W. (2021). Dermoscopy of Small Diameter Melanomas with the Diagnostic Feasibility of Selected Algorithms—A Clinical Retrospective Multicenter Study. Cancers.

[31] Hunziker M.F.V., Abdalla B.M.Z., Brandão F.V., Meneghello L.P., Hunnicutt J.M.S., Di Giacomo T.H.B., Abdalla C.M.Z., Sortino A.M.F. (2023). Exploring Small-Diameter Melanomas: A Retrospective Study on Clinical and Dermoscopic Features. Life.

[32] Purim K.S.M., de Santana Avelar M.F. (2012). [Photoprotection, Melasma and Quality of Life in Pregnant Women]. Rev. Bras. Ginecol. Obs..

[33] Roh M.R., Eliades P., Gupta S., Tsao H. (2015). Genetics of Melanocytic Nevi. Pigment Cell Melanoma Res..

[34] Yeh I. (2020). New and Evolving Concepts of Melanocytic Nevi and Melanocytomas. Mod. Pathol..

[35] Bieber A.K., Martires K.J., Driscoll M.S., Grant-Kels J.M., Pomeranz M.K., Stein J.A. (2016). Nevi and Pregnancy. J. Am. Acad. Dermatol..

[36] Born L.J., Tembunde Y., Driscoll M.S., Grant-Kels J.M. (2025). Melanoma and Melanocytic Nevi in Pregnancy. Clin. Dermatol..

[37] Amaral T., Ottaviano M., Arance A., Blank C., Chiarion-Sileni V., Donia M., Dummer R., Garbe C., Gershenwald J.E., Gogas H. (2025). Cutaneous Melanoma: ESMO Clinical Practice Guideline for Diagnosis, Treatment and Follow-Up. Ann. Oncol..

[38] Swetter S.M., Tsao H., Bichakjian C.K., Curiel-Lewandrowski C., Elder D.E., Gershenwald J.E., Guild V., Grant-Kels J.M., Halpern A.C., Johnson T.M. (2019). Guidelines of Care for the Management of Primary Cutaneous Melanoma. J. Am. Acad. Dermatol..

[39] Nading M.A., Nanney L.B., Boyd A.S., Ellis D.L. (2008). Estrogen Receptor Beta Expression in Nevi during Pregnancy. Exp. Dermatol..

[40] Dika E., Patrizi A., Lambertini M., Manuelpillai N., Fiorentino M., Altimari A., Ferracin M., Lauriola M., Fabbri E., Campione E. (2019). Estrogen Receptors and Melanoma: A Review. Cells.

[41] Wyon Y., Synnerstad I., Fredrikson M., Rosdahl I. (2007). Spectrophotometric Analysis of Melanocytic Naevi during Pregnancy. Acta Derm. Venereol..

[42] Driscoll M.S., Grant-Kels J.M. (2007). Hormones, Nevi, and Melanoma: An Approach to the Patient. J. Am. Acad. Dermatol..

[43] Taglialatela I., Indini A., Santanelli G., Di Liberti G., Di Guardo L., De Braud F., Del Vecchio M. (2024). Melanoma and Sex Hormones: Pathogenesis, Progressive Disease and Response to Treatments. Tumori J..

[44] Carter T.J., George C., Harwood C., Nathan P. (2022). Melanoma in Pregnancy: Diagnosis and Management in Early-Stage and Advanced Disease. Eur. J. Cancer.

[45] Martins-Costa G.M., Bakos R. (2019). Total Body Photography and Sequential Digital Dermoscopy in Pregnant Women. Dermatol. Pr. Concept..

[46] Strumia R. (2002). Digital Epiluminescence Microscopy in Nevi during Pregnancy. Dermatology.

[47] Pennoyer J.W., Grin C.M., Driscoll M.S., Dry S.M., Walsh S.J., Gelineau J.P., Grant-Kels J.M. (1997). Changes in Size of Melanocytic Nevi during Pregnancy. J. Am. Acad. Dermatol..

[48] Zampino M.R., Corazza M., Costantino D., Mollica G., Virgili A. (2006). Are Melanocytic Nevi Influenced by Pregnancy? A Dermoscopic Evaluation. Dermatol. Surg..

[49] Peter J.K., Helfenstein F., Cerminara S.E., Maul J.T., Zehnder M.L., Jamiolkowski D., Roider E., Mühleisen B., Hösli I., Navarini A.A. (2025). AI-Assisted Total Body Dermoscopic Evaluation of Changes in Melanocytic Nevi during Pregnancy: A Prospective, Comparative Study of 2,799 Nevi. Acta Derm. Venereol..

[50] Aktürk A.S., Bilen N., Bayrämgürler D., Demirsoy E.O., Erdogan S., Kiran R. (2007). Dermoscopy Is a Suitable Method for the Observation of the Pregnancy-Related Changes in Melanocytic Nevi. J. Eur. Acad. Dermatol. Venereol..

[51] Ellis D.L. (1991). Pregnancy and Sex Steroid Hormone Effects on Nevi of Patients with the Dysplastic Nevus Syndrome. J. Am. Acad. Dermatol..

[52] Cosgarea I., Trevisan-Herraz M., Ungureanu L., Zalaudek I. (2021). Dermatoscopic Features of Naevi During Pregnancy—A Mini Review. Front. Med..

[53] Rubegni P., Sbano P., Burroni M., Cevenini G., Bocchi C., Severi F.M., Risulo M., Petraglia F., Dell’eva G., Fimiani M. (2007). Melanocytic Skin Lesions and Pregnancy: Digital Dermoscopy Analysis. Ski. Res. Technol..

[54] Friedman E.B., Scolyer R.A., Thompson J.F. (2019). Management of Pigmented Skin Lesions during Pregnancy. Aust. J. Gen. Pr..

[55] Zampetti A., Feliciani C., Landi F., Capaldo M.L., Rotoli M., Amerio P.L. (2006). Management and Dermoscopy of Fast-Growing Nevi in Pregnancy: Case Report and Literature Review. J. Cutan. Med. Surg..

[56] What to Look for: ABCDEs of Melanoma. https://www.aad.org/public/diseases/skin-cancer/find/at-risk/abcdes.

[57] Proietti I., Skroza N., Michelini S., Mambrin A., Balduzzi V., Bernardini N., Marchesiello A., Tolino E., Volpe S., Maddalena P. (2020). BRAF Inhibitors: Molecular Targeting and Immunomodulatory Actions. Cancers.

[58] Lorenzi E., Simonelli M., Persico P., Dipasquale A., Santoro A. (2021). Risks of Molecular Targeted Therapies to Fertility and Safety during Pregnancy: A Review of Current Knowledge and Future Needs. Expert Opin. Drug Saf..

[59] Hassel J.C., Livingstone E., Allam J.P., Behre H.M., Bojunga J., Klein H.H., Landsberg J., Nawroth F., Schüring A., Susok L. (2021). Fertility Preservation and Management of Pregnancy in Melanoma Patients Requiring Systemic Therapy. ESMO Open.

[60] Maleka A., Enblad G., Sjörs G., Lindqvist A., Ullenhag G.J. (2013). Treatment of Metastatic Malignant Melanoma with Vemurafenib during Pregnancy. J. Clin. Oncol..

[61] De Haan J., Van Thienen J.V., Casaer M., Hannivoort R.A., Van Calsteren K., Van Tuyl M., Van Gerwen M.M., Debeer A., Amant F., Painter R.C. (2018). Severe Adverse Reaction to Vemurafenib in a Pregnant Woman with Metastatic Melanoma. Case Rep. Oncol..

[62] Marcé D., Cornillier H., Denis C., Jonville-Bera A.P., Machet L. (2019). Partial Response of Metastatic Melanoma to BRAF-Inhibitor-Monotherapy in a Pregnant Patient with No Fetal Toxicity. Melanoma Res..

[63] Pagan M., Jinks H., Sewell M. (2019). Treatment of Metastatic Malignant Melanoma during Pregnancy with a BRAF Kinase Inhibitor. Case Rep. Womens Health.

[64] Ott P.A., Hodi F.S., Robert C. (2013). CTLA-4 and PD-1/PD-L1 Blockade: New Immunotherapeutic Modalities with Durable Clinical Benefit in Melanoma Patients. Clin. Cancer Res..

[65] Wang S., Chen C., Li M., Qian J., Sun F., Li Y., Yu M., Wang M., Zang X., Zhu R. (2019). Blockade of CTLA-4 and Tim-3 Pathways Induces Fetal Loss with Altered Cytokine Profiles by Decidual CD4+T Cells. Cell Death Dis..

[66] Selby M.J., Engelhardt J.J., Johnston R.J., Lu L.S., Han M., Thudium K., Yao D., Quigley M., Valle J., Wang C. (2016). Preclinical Development of Ipilimumab and Nivolumab Combination Immunotherapy: Mouse Tumor Models, In Vitro Functional Studies, and Cynomolgus Macaque Toxicology. PLoS ONE.

[67] Poulet F.M., Wolf J.J., Herzyk D.J., Degeorge J.J. (2016). An Evaluation of the Impact of PD-1 Pathway Blockade on Reproductive Safety of Therapeutic PD-1 Inhibitors. Birth Defects Res. B Dev. Reprod. Toxicol..

[68] Andrikopoulou A., Korakiti A.M., Apostolidou K., Dimopoulos M.A., Zagouri F. (2021). Immune Checkpoint Inhibitor Administration during Pregnancy: A Case Series. ESMO Open.

[69] Anami Y., Minami S., Kumegawa A., Matsukawa H., Nishioka K., Noguchi T., Iwahashi N., Mizoguchi M., Nanjo S., Ota N. (2021). Malignant Melanoma Treated with Pembrolizumab during Pregnancy: A Case Report and Review of the Literature. Mol. Clin. Oncol..

[70] FDA IMLYGIC. https://www.fda.gov/vaccines-blood-biologics/cellular-gene-therapy-products/imlygic.

[71] FDA, Cder PROLEUKIN ® (Aldesleukin) for Injection, for Intravenous Infusion Rx Only. https://www.accessdata.fda.gov/drugsatfda_docs/label/2012/103293s5130lbl.pdf.

